# Analysis of wear, wear particles, and reduced inflammatory potential of vitamin E ultrahigh-molecular-weight polyethylene for use in total joint replacement

**DOI:** 10.1002/jbm.b.32904

**Published:** 2013-02-22

**Authors:** C L Bladen, S Teramura, S L Russell, K Fujiwara, J Fisher, E Ingham, N Tomita, J L Tipper

**Affiliations:** 1 Institute of Medical and Biological EngineeringUniversity of Leeds, UK; 2 Graduate School of EngineeringKyoto University, Japan; 3 R&D Centre, Nakashima Medical Co. LtdJapan

**Keywords:** vitamin E, UHMWPE, oxidative damage, oxidative stress, osteolytic mediators, novel bearing materials

## Abstract

Vitamin E (VE) has been added to ultrahigh-molecular-weight polyethylene (UHMWPE) acetabular cups and tibial trays primarily to reduce oxidative damage to the polymer. The aim of this study was to investigate the relative wear rates of UHMWPE-containing VE compared with virgin UHMWPE. The ability of VE to reduce the amount of inflammatory cytokines produced from stimulated peripheral blood mononuclear cells (PBMNCs) was also investigated. Stimulation was achieved by exposure of PBMNCs to either lipoplysaccharide (LPS) or VE-containing UHMWPE (VE-UHMWPE). In the present study, results showed that the wear rates of UHMWPE with or without VE were not significantly different. Particles generated by UHMWPE with and without VE were not significantly different in size distribution. The production of osteolytic mediators, tumor necrosis factor-alpha, interleukin 1β (IL-β), IL-6, and IL-8 were significantly reduced in (PBMNCs) stimulated with either LPS + VE compared with LPS or VE-UHMWPE particles compared to virgin UHMWPE particles. This trend was also observed when VE was added as a liquid to UHMWPE wear particle-stimulated PBMNCs. The exact mechanism of how VE affects the release of inflammatory mediators from particle-stimulated macrophages is not yet understood. It is likely to involve the anti-inflammatory and/or antioxidant effects of VE.

## INTRODUCTION

Total hip replacement (THR) is a successful surgical intervention, eliminating pain, increasing mobility, and restoring quality of life. Hip replacement is the most common joint replacement procedure comprising almost 80% of total joint surgeries.^1^ There are several bearing material choices available [metal-on-polyethylene (MPE), metal-on-metal, metal-on-ceramic, and ceramic-on-ceramic]. Prostheses comprising an acetabular component of highly cross-linked ultrahigh-molecular-weight polyethylene (UHMWPE) are the most frequently used worldwide. While THR is a broadly successful procedure, over a period of 10–15 years, a significant proportion (10–20%) will fail particularly in younger patients (<65 years old), who will often outlive the working life of the implant. This is generally due to patient specific factors, for example, age at implantation, activity level,^2^, ^3^ surgical technique, fixation of the implant to the bone, osteolysis, and long-term bone remodelling, with 75% of failures being attributable to aseptic loosening due to osteolysis. With conventional MPE, it is believed that the major cause of osteolysis and subsequent aseptic loosening is due to the biological response to UHMWPE wear debris generated at the articulating interface, mainly by the interaction of macrophages with these particles.^1^ Highly cross-linked UHMWPE offers significant advantages over conventional UHMWPE in terms of reduced osteolytic potential.^4^ However, failure does still occur in highly cross-linked UHMWPE, in particular there is an increase in rim fracture due to a decrease in fatigue resistance.^5^, ^6^

The size range of UHMWPE wear particles can vary from nanometers up to several millimeters.^7^ It is now clear that particles in the size range of 0.1–1 μm are the most biologically reactive.^8^, ^9^ In addition to the size, the volume of wear debris plays an important role in the biological reactivity of the particles.^8^, ^9^

The biological response to UHMWPE wear debris is dependent on the volume of particles within a critical size range.^8^ It has been found that only particle volume to cell number ratios of Œ10 μm^3^/cell is capable of eliciting a significant biological response to UHMWPE particles, in terms of osteolytic cytokine release.^9^ Analysis of periprosthetic tissues retrieved from revision surgeries has demonstrated the presence of biochemical mediators of inflammation that are associated with macrophage activation. Cytokines that have been detected in the periprosthetic tissues include TNF-α (tumor necrosis factor-alpha), IL-1β (interleukin 1β), IL-6, IL-8, IL-11, macrophage colony stimulating factor CSF, granulocyte-macrophage CSF, transforming growth factor-α and –β, and prostaglandin E_2_. TNF-α is the most abundantly produced cytokine and has become a widely accepted marker for inflammation.^10^

The process by which UHMWPE implants are manufactured also contributes to prosthesis failure, mainly due to oxidation-related problems with the polymer and the generation of free radicals and other free oxygen species. Oxidation of the polymer may arise due to the processes of compression molding, postirradation treatment, storage and sterilization, (γ-irradiation in air or inert atmosphere).

Vitamin E (VE; α-tocopherol) was first introduced into conventional UHMWPE and highly cross-linked UHMWPE in an attempt to decrease surface delamination caused by oxidative fatigue. VE is a free radical scavenger and well established biological antioxidant. It is a naturally occurring compound that functions to maintain the long-chain polyunsaturated fatty acid in the cell membrane.^11^ By incorporating a lipophilic and biocompatible antioxidant into UHMWPE, it was thought that the adverse effects of oxidation-induced free radicals on the mechanical integrity of this type of bearing material would be decreased. VE was added at 3000 ppm to conventional and cross-linked UHMWPE and it was reported that VE prevented oxidation and delamination and had a favorable long-term effect on fatigue performance of the UHMWPE, increasing the resistance to fatigue cracks that are associated with oxidation,^12^ without negatively altering the biocompatibility of the implant.^13^ The effect of VE on wear debris size and morphology has been recently investigated in the knee.^14^ UHMWPE was formulated with 3000 ppm VE by direct compression molding and tested in a multidirectional knee simulator. The results demonstrated differences between virgin and 3000 ppm VE UHMWPE in terms of wear volume, in favor of the VE-containing formulation. The wear volume was reduced by 45% and there was also a reduction in the proportion of wear particles in the submicrometer size range for VE UHMWPE, which are the most biologically active in terms of cytokine release.^14^ VE-blended UHMWPE has been marketed for knee joint inserts as BLEND-E in Japan since February 2010. Blend-E inserts have been used in about 2000 patients and have been *in situ* for 2.5 years. Only one joint has had to be retrieved due to infection. Blend-E was manufactured via direct compression molding following the blending of UHMWPE resin powder (GUR1050, Ticona) with VE (dl-a-tocopherol; Eisai Co., Japan) at 0.3 wt %. Blend-E is sterilized with ethylene oxide gas. Studies confirmed that VE exists uniformly by measuring Tocopherol index by Fourier transform infrared spectroscopy (FT-IR; in-house data from Japan). Previous studies on highly cross-linked VE-containing UHMWPE (VE-UHMWPE) in the hip have shown a decrease in wear; however, it is not clear if this is due to the VE or the cross-linking itself.^15^

VE is well characterised and well used as an antioxidant in many nonorthopeadic disease states and recently, the anti-inflammatory effects of VE have been investigated in conditions such as atherosclerosis.^16^ As in THR-associated osteolysis, TNF-α has pleiotropic biological actions in atherosclerosis. The anti-inflammatory effects of VE were achieved *in vivo* using a high dose of oral VE (1200 IU/day).^17^ Volunteers were supplemented with 1200 IU/day of VE for 8 weeks, after which their monocytes activated with lipoplysaccharide (LPS), produced lower quantities of TNF-α compared to control monocytes from individuals not supplemented with VE.

The aim of the study was to investigate the effects of VE on the wear rates and particle size distribution of noncross-linked VE-containing GUR1050 UHMWPE compared to virgin GUR1050. In addition, the anti-inflammatory effects of different concentrations of VE (see Table I) on LPS and UHMWPE-stimulated PBMNCs were studied.

**I:** Materials Used in the Study and Tests Performed

Experimental Materials	Treatments	Clinical Material	Treatments	Liquid Vitamin E	Treatments
Virgin GUR1050	Wear test analysis. Stimulation of PBMNCs with Virgin GUR1050 versus GUR1050 + 3000 ppm VE and GUR1050 + 30,000 ppm VE	Virgin GUR1050	Stimulation of PBMNCs with virgin GUR1050 or LPS versus GUR1050 + 1000 ppm VE	dl-α-Tocopherol acetate	Dose response analysis. Stimulation of PBMNCs with LPS followed by addition of liquid VE
GUR1050 + 3000 ppm VE (Blend E in Japan)		GUR1050 + 1000 ppm VE			
GUR1050 + 30,000 ppm VE					

## MATERIALS AND METHODS

Table I gives an outline of the different materials used in the present study and the tests performed on the various materials.

Biologically relevant wear particles were generated in a multidirectional single-station wear rig in order to produce large volumes of sterile wear particles that could be added aseptically to cells in culture. These particles have been shown to be comparable to “real” wear particles produced in the six-station wear simulator and those generated *in vivo*.^7^, ^18^ The particles were shown to be sterile and endotoxin free. The particles were cultured with peripheral blood mononuclear cells (PBMNCs) with appropriate positive and negative controls and with and without the addition of VE. Biological activity of the particles was determined by relative cytokine release (compared to controls). All experiments were carried out using PBMNCs from three healthy donors and were repeated three times with cells for each donor.

### UHMWPE pin production

#### Experimental materials

UHMWPE pins were consolidated using a process previously reported by Teramura et al.^14^

#### Clinical material

GUR1050 UHMWPE-containing VE at 1000 ppm was a gift from Meditech Medical Polymers. The UHMWPE pins were machined from a single block of material.

All materials were nonirradiated. All pins were machined to form a truncated cone with a flat surface of 8 mm diameter, which formed the contact face. The pins were soaked in deionised water (standard operating procedure, IMBE) for a minimum of 2 weeks prior to testing in order to stabilise their water content (necessary with nonirradiated UHMWPE).^19^

### Wear test evaluation

A multidirectional six-station pin-on-plate wear simulator was used to determine the wear rates of UHMWPE with and without VE. Protocol was a modified version of Galvin et al.^20^ The wear rate was evaluated using a six-station wear simulator against smooth CoCr plates (*R*_a_ 0.01–0.03 µm). The pin was loaded under a constant force of 160 N (nominal contact pressure of 3.2 MPa) and the plate was moved in a reciprocating motion underneath the pin. Four pins of each UHMWPE were tested. The lubricant was 25% (v/v) bovine serum, diluted using 0.1% (w/v) sodium azide. The pins were weighed prior to the start of the test and at the end of each week of testing. The mass loss was converted to volume loss and the wear factor (*k*) was calculated according to Ref. 21.

Endotoxin-free aseptic UHMWPE wear debris was generated using a multidirectional single-station wear simulator, according to an established protocol.^22^ Particles for cell culture studies were generated from GUR1050 UHMWPE (virgin), GUR1050 UHMWPE-containing 1000 ppm VE, GUR1050 UHMWPE-containing 3000 ppm VE, and GUR1050 UHMWPE-containing 30,000 ppm VE. All materials were nonirradiated.

### Isolation and analysis of UHMWPE wear debris from six-station pin on plate wear tests

Wear test lubricants were collected at the end of each week of the wear tests and stored at −20°C. The 25% (v/v) bovine serum lubricant was subsequently digested as described previously Richards et al.^7^

### Isolation and analysis of UHMWPE wear debris from the single-station wear rig

Wear particles were also isolated from the 25% (v/v) foetal bovine serum lubricants from the aseptic wear tests using the same method described above Richards et al.^7^ Over 600 particles were analyzed for each sample from randomly selected areas of the filters. In the single-station wear rig, the wear particles were also isolated from the 25% (v/v) feotal bovine serum lubricants from the aseptic wear tests using the same method described above and frequency and area distributions were obtained for each UHMWPE material. This allowed the authors to be confident that the particles generated under aseptic conditions for the cell culture studies were of a similar size and shape to those generated in the wear studies.

### Microbiological assessments of aseptic wear test lubricants

Samples of lubricant were taken from all aseptic wear tests at the start and end of each test, and plated onto nutrient agar (NA), heated blood agar (HBA), and Saboraud’s dextrose agar (SAB; Unipath, UK). The NA and HBA plates were incubated at 37°C, and the SAB plates at 30°C, for up to 48 h with inspection every 24 h in the presence of microbial contamination. Only particles in lubricants that had no microbiological contamination was used for the cell culture studies.

### Measurement of endotoxin concentration in aseptic wear test lubricants (LAL kinetic-QCL, endotoxin assay)

The limulus amebocyte lysate (LAL) endotoxin assay (Lonza) was used to determine the presence of endotoxin in all particle lubricants prior to cell culture studies. Samples were diluted 1:200 using endotoxin free water (Cambrex), and the assay was carried out according to manufacturer’s guidelines.

### VE stock solution (added as liquid for cell studies)

VE was provided as dl-α-tocopherol acetate at a concentration of 500 mg/mL (Merck, Germany). A 20-m*M* stock solution of VE was prepared in Roswell Park Memorial Institute medium (RPMI) 1640 cell culture medium and serial dilutions were made to achieve working a concentration of (800 µ*M*).

### Culture of primary human PBMNCs with UHMWPE wear particles (experimental and clinical materials)

Ethical approval for use of human volunteers was granted by University of Leeds, Faculty of Biological Sciences ethics committee. PBMNCs were isolated from whole heparinised blood from healthy volunteers using lymphoprep™ gradients (Axis-Shield, Norway). Mononulear cells were seeded into 24-well tissue culture plates at a seeding density of 2 × 10^5^ cells/well in RPMI 1640 medium supplemented with 10% (v/v) foetal bovine serum and antibiotics (penicillin and streptomycin 100 µg/mL). In the case of UHMWPE (with or without VE), the particles were placed in 1.5% (w/v) low melting temperature agarose prior to cell seeding, since the particles float in solution. UHMWPE particles were dosed at either 10 µm^3^ particles/cell or 100 µm^3^ particles/cell.^23^ Where VE was added separately in solution, a dose of 800 µ*M* was used immediately after cell seeding (unpublished in-house data where dose is nontoxic but does produce anti-inflammatory effects). Cell viability (ATPlite™, Perkin Elmer) and cytokine production (TNF-α, IL-1β, IL-6, and IL-8), determined using Enzyme-linked immunosorbent assay (ELISA; R&D Systems) were measured at 12 and 24 h or just at 24 h postcell seeding. In all experiments, a cells only negative control and LPS (200 ng/mL) positive control were used.

### LPS stimulation of PBMNCs followed by addition of VE as a liquid

PBMNCs were stimulated with lipopolysaccharide (LPS) at a concentration of 200 ng/mL. VE was then added at a dose of 800 µ*M* to see if VE has an effect on the amount of TNF-α produced by the LPS-stimulated PBMNCs. Treatments were as follows: LPS added at time 0 followed by addition of VE at +3 h, LPS and VE both added at time 0, or VE added at time 0 followed by addition of LPS at +3 h. Cell viability assays (ATP-Lite™) and ELISA for TNF-α were performed as described above after 24 h incubation at 37°C in an atmosphere of 5% (v/v) CO_2_ in air.

### Statistical analysis

The results were expressed as the mean value ± 95% confidence limits (*n* = 4). Since percentage data does not exhibit a normal distribution, it was necessary to arcsine transform and then back transform the data for illustrative purposes. All results were compared by two-way analysis of variance (ANOVA) followed by a Bonferroni post-test to allow for multiple comparisons. Statistical significance was considered at *p* < 0.05.

## RESULTS

### Wear test evaluation and analysis

**FIGURE 1:**
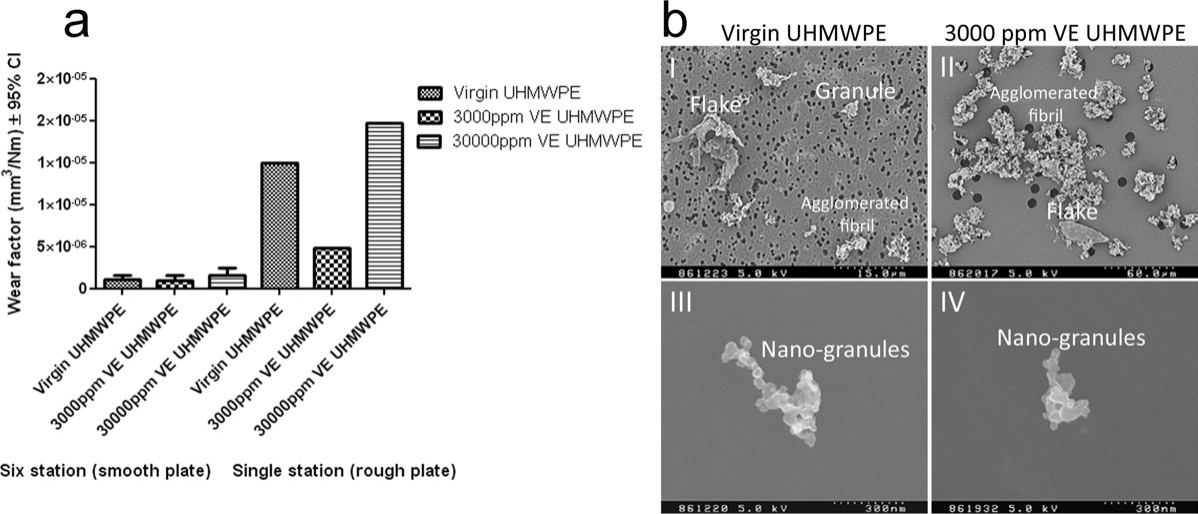
Wear and wear particle analysis of UHMWPE with and without Vitamin E. (a) Wear factors for virgin UHMWPE and UHMWPE with 3000 ppm and 30,000 ppm vitamin E against smooth and rough CoCr plates in the six-station and single-station wear rigs, respectively. (b) High-resolution FEGSEM images of UHMWPE particles from (I) virgin UHMWPE showing granule, flake, and agglomerated fibril particle morphologies on the 1 µm filter, size bar 15 µm. (II) Particles from the 3000 ppm vitamin E UHMWPE showing flake and agglomerated fibril morphologies on the 10 µm filter, size bar 60 µm. (III) Nanometer-sized granules from virgin UHMWPE on the 0.015 µm filter, size bar 300 nm. Nanometer-sized granules (IV) from the 3000 ppm VE UHMWPE material, size bar 300 nm.

Wear test evaluations of the *experimental materials*-containing VE compared to virgin UHMWPE (without VE) were carried out using a six-station simple configuration multidirectional wear simulator. There were no significant differences between the wear rates of the three UHMWPE materials when tested using the six-station pin-on-plate wear simulator against a smooth counterface Figure 1(a). The UHMWPE-containing 30,000 ppm VE had a slightly higher, but not significant wear rate compared to the virgin UHMWPE and the UHMWPE-containing 3000 ppm VE. Wear rate data cannot be included from the single-station simulator as it cannot be tested statistically since *n* = 1, although in this particular test a reduction in wear rate was observed for the UHMWPE-containing 3000 ppm VE. The single-station simulator simply allows for the production large volumes of sterile wear debris. The wear particles were isolated from the wear test lubricants from the three materials by alkaline digestion and displayed a range of similar morphologies including fibrils, flakes and granules Figure 1(b). The majority of the particles were submicrometer sized granules.

**FIGURE 2:**
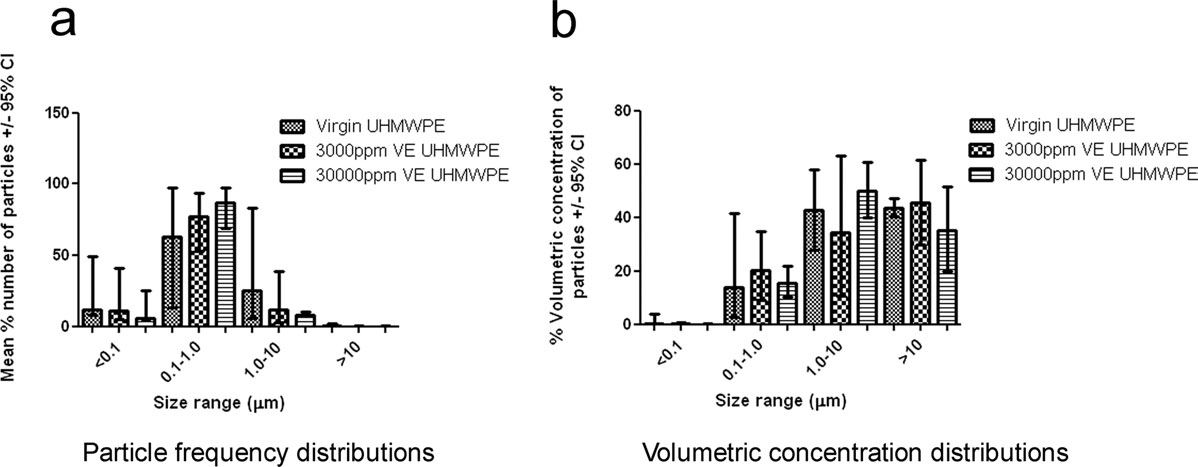
Particle frequency distributions and volumetric concentration distributions for UHMWPE with and without Vitamin E from wear simulator. (a) UHMWPE particle frequency distributions from the six-station wear rig using smooth CoCr plates (*n* = 4). (b) volumetric concentration distributions for particles from the six-station wear rig against smooth CoCr plates (*n* = 4).

Wear debris analysis from the six-station pin-on-plate wear simulator showed that the wear particles generated by all three materials had similar frequency distributions, with the mode of the distributions falling in the 0.1–1.0 µm size range for all three materials Figure 2(a). Wear rate data cannot be included from the single-station simulator as it cannot be tested statistically since *n* = 1. There were no significant differences between the particle frequency distributions generated by the virgin UHMWPE and the UHMWPEs that contained VE. The volumetric concentration distributions of the wear particles were more variable, with the mode of the distributions falling in either the 1–10 µm size range (30,000 ppm VE) or the >10 µm size range (virgin UHMWPE and 3000 ppm VE Figure 2(b). There were no significant differences in the particle volume distributions between the three materials. The volume distributions demonstrated that a small number of particles in the >10 µm size range accounted for a large proportion of the total wear volume Figure 2(a,b). Wear particles were also isolated from the single-station wear test lubricants (rough counterfaces) from the three materials. The wear particles demonstrated similar size and volume distributions to those isolated from the six-station pin on plate wear tests (smooth counterfaces), data not shown.

### Generation of sterile wear particles from experiments materials and analysis of cytokine release

**FIGURE 3:**
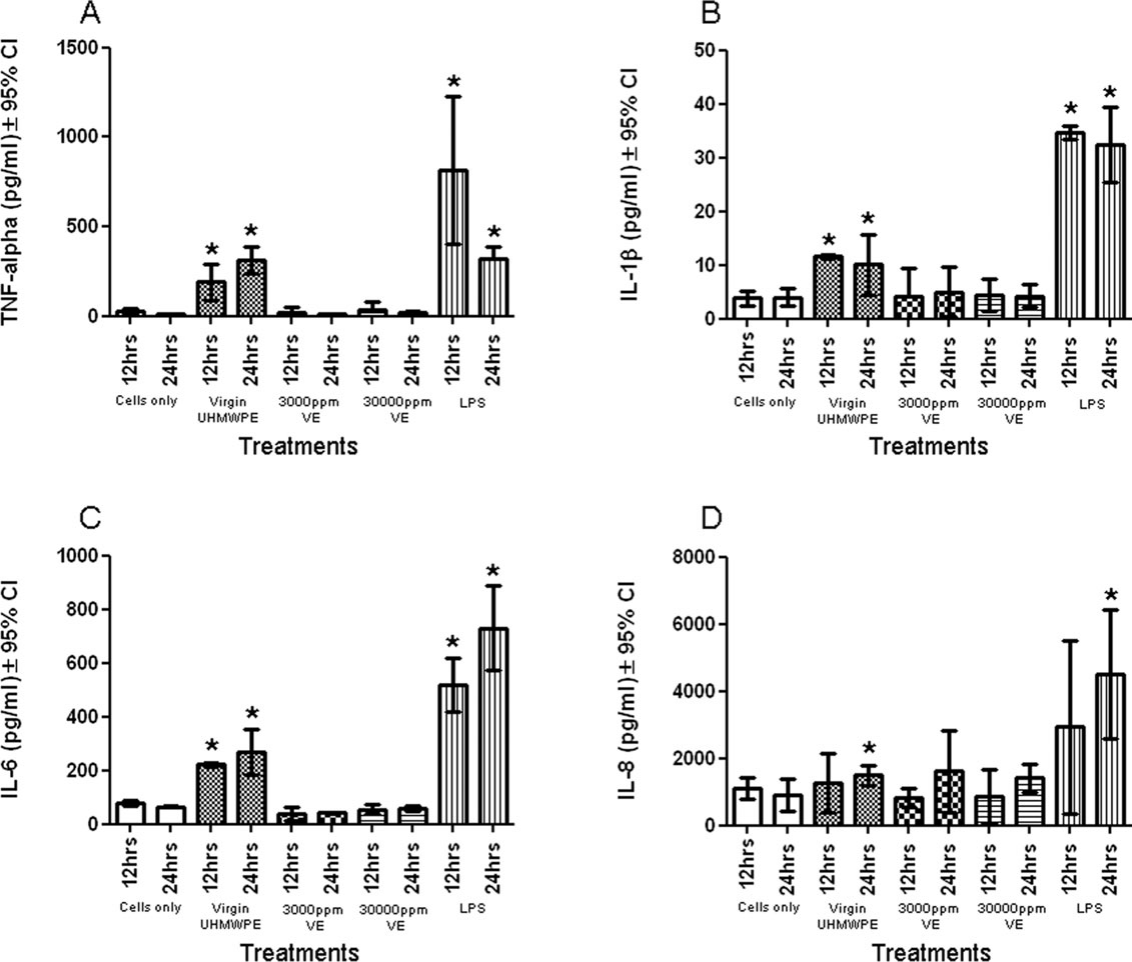
Cytokine production from human PBMNCs cultured with UHMWPE particles with and without vitamin E. (a) TNF-α release. (b) IL-1β release. (c) IL-6 release. (d) IL-8 release. PMNBCs were cultured with particles from virgin UHMWPE, 3000 ppm VE, and 30,000 ppm VE and cytokine production measured at 12 and 24 h by ELISA. LPS (200 ng/mL) was added as a positive control. Cells only provided a negative control. Three donors were analyzed for each cytokine. Results are expressed for one donor as mean ± 95% CL for the four replicate wells. All results were compared by two-way analysis of variance followed by a bonferroni post-test to allow for multiple comparisons. * indicates a statistically significant difference compared to the cells only negative control (*p* < 0.05; ANOVA).

Aseptic wear particles were generated from the *experimental materials* (virgin UHMWPE, 3000 ppm and the 30,000 ppm VE) and cultured *in vitro* with PBMNCs from three donors. PBMNCs cultured with 100 µm^3^ particles/cell^23^ from virgin UHMWPE caused the release of significantly higher quantities of TNF-α at 12 h (*p* = 0.0326) and 24 h (*p* = 0.0018), compared to wear particles from both the 3000 ppm and the 30,000 ppm VE UHMWPE, which both caused the release of levels of TNF-α comparable to the cells with no particles [cell only control group; Figure 3(a)]. The results for the other cytokines, IL-1β and IL-6 showed similar trends, with the wear particles from the virgin UHMWPE material consistently producing significantly higher quantities of cytokine than the particles from the VE enhanced UHMWPEs (Figure 3(b,c). However, significantly higher levels of IL-8 were only released by PBMNCs from one out of the three donors at the 24 h time point. The results for the cells from two additional donors showed similar trends as those shown in Figure 3 (data not shown).

### VE dose response data

**FIGURE 4:**
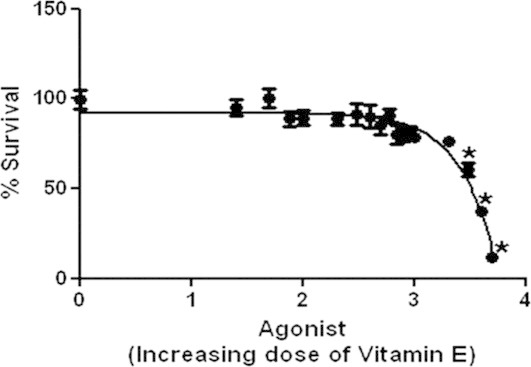
Vitamin E dose response curve. The effect of increasing dose of vitamin E (0 µ*M* to 5 m*M*) on PBMNC viability was investigated. Data was fitted to a sigmoidal dose response curve by log transforming the X column values then normalizing the Y column values. A nonlinear regression was then performed prior to fitting the data to a sigmoidal dose response curve with a variable slope. Results represent average of triplicates from a single donor.

Prior to experiments with the *clinical material* (1000 ppm VE), the cytotoxicity of VE was assessed in PBMNCs using the ATPlite™ assay and adverse effects were only observed at relatively high doses Figure 4, >3 m*M* (>3000 ppm). VE dose response curves were repeated a minimum of three times with cells from three different donors (representative data shown). It was established that VE at a dose of 800 µ*M* was optimal for use in the *in vitro* experiments, and that this dose was not cytotoxic but showed anti-inflammatory effects.

**FIGURE 5:**
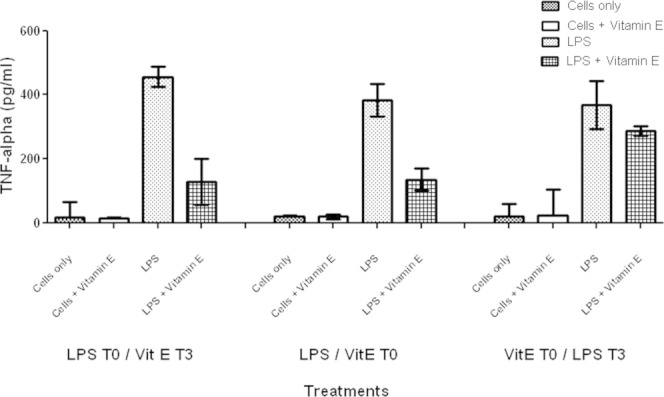
Moderation of TNF-α production by vitamin E (added as a liquid) in PBMNCs stimulated with LPS (lipopolysaccharide; 200 ng/mL). Treatments were as follows; LPS at time 0 followed by VE (800 µ*M*) at +3 h, LPS and VE at time 0, or VE at time 0 followed by LPS at +3 h. Results show mean ± 95% confidence limits, * indicates a statistically significant difference compared to cells only negative control, ∧ indicates significant reduction in LPS-stimulated TNF-α release after vitamin E addition (*p* < 0.05; ANOVA). Results represent average of triplicates from a single donor.

When VE was added separately as a liquid to mimic VE supplementation (800 µ*M*) the response of LPS-stimulated PBMNCs was modulated and lower levels of TNF-α were produced compared to control LPS-stimulated PBMNCs (Figure 5). This modulation was significant when VE was added either 3 h after initial LPS stimulation (LPS T0/VitE T3, *p* = 0.0161) or at the same time as LPS addition (LPS/VitE T0, *p* = 0.0030). Addition of VE 3 h prior to LPS stimulation (VitE T0/Ltfmk PS T3, ns) did not significantly reduce the amount of TNF-α produced, suggesting that VE does not function in a pretreatment protective manner (Figure 5).

### Generation of sterile wear particles from clinical material and analysis of TNF-α release

**FIGURE 6:**
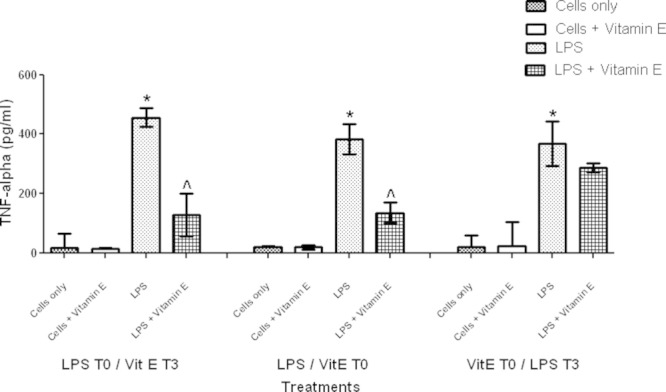
TNF-α production in PBMNCs after stimulation with UHMWPE particles from the clinical material (1000 ppm VE). (a) the effect of clinically relevant GUR1050 UHMWPE wear particles on TNF-α production in the presence and absence of vitamin E added as a liquid (800 µ*M*). (b) the effect of clinically relevant wear particles containing 1000 ppm vitamin E (PVE) on TNF-α production in PBMNCs. Particles were dosed at 10 µm^3^/cell (10:1) and 100 µm^3^/cell (100:1). Results show mean ± 95% confidence limits, * indicates a statistically significant difference compared to cells only negative control. + indicates significant reduction in TNF-α released in UHMWPE stimulated PBMNCs after vitamin E addition. ∧ indicates significant reduction in LPS stimulated TNF-α release after vitamin E addition (*p* < 0.05; ANOVA). Results represent average of triplicates from a single donor.

*Clinically relevant wear particles* were generated aseptically from GUR1050 UHMWPE-containing VE at 1000 ppm, using a single-station wear test simulator. Clinically relevant UHMWPE (GUR1050) particles without VE at a dose of 100 µm^3^/cell^23^ stimulated the production of significantly elevated levels of TNF-α (*p* = 0.0156) compared to the cells only negative control and virgin UHMWPE at a dose of 10 µm^3^ Figure 6(a). There was a significant reduction (*p* = 0.0270) in TNF-α production in the presence of UHMWPE particles with 800 µ*M* VE, added separately Figure 6(a). Addition of VE solution was at T0. When particles generated from the *clinically relevant* polyethylene-containing 1000 ppm vitamin E (PVE) were used to stimulate PBMNCs at 100 µm^3^/cell,^23^ the production of TNF-α was significantly reduced (*p* = 0.0073, *p* = 0.0008) compared to stimulation by either GUR1050 UHMWPE wear particles or LPS controls Figure 6(b). These experiments were repeated with cells from three different donors and similar results were obtained (representative data shown). Cell viability was not affected by any of the treatments (data not shown). LPS stimulated (200 ng/mL) PBMNCs released significantly elevated levels of TNF-a compared to the negative cell only controls, and once again the presence of VE (800 µ*M*) significantly modulated TNF-α production (*p* = 0.0270; *p* = 0.0073; Figures 6(a) and 5(b), respectively.

## DISCUSSION

UHMWPE-containing VE has been shown to have a lower wear rate in hip and knee simulator studies.^14^, ^15^, ^22^, ^24^ However, in the present study the wear rates of VE-containing UHMWPEs in the six-station simulator were not significantly different from the virgin UHMWPE. In the case of the single-station wear rig, we did see a lower wear rate for the UHMWPE-containing 3000 ppm VE but these data are not statistically robust. Previous studies have shown a reduction in wear rate with the addition of VE to highly cross-linked UHMWPE compared to conventional UHMWPE but in many of the studies, it is not clear whether the reduction in wear rate was due to the addition of VE or the high levels of cross-linking.^22^, ^25^ In the present study, the addition of VE to non irradiated UHMWPE did not affect the wear factor suggesting that in previous studies, the reduction in wear was more likely to be associated with the high levels of cross-linking. The biological response to wear particles *in vitro* is strongly influenced by both particle size and volume.^8^ The wear particles isolated from the VE-containing UHMWPEs were not significantly different in size distribution or volume distribution compared to the virgin material, however, the wear debris generated from both UHMWPEs containing VE had reduced biological activity when cultured with PBMNCs from several different donors. Wear particles from the VE-UHMWPE materials consistently elicited significantly reduced levels of TNF-α, IL-1β, and IL-6 from PBMNCs at the 12 h and 24 h time points. PBMNCs stimulated with LPS produced lower amounts of TNF-alpha when VE was present. This was true if VE was added 3 h after the addition of LPS or if VE was added at the same time as the LPS. Pretreatment with VE did not offer a protective effect to the cells later stimulated with LPS.

A detailed mechanism for the reduced cellular response to debris from UHMWPE-containing VE has yet to be determined. However, a reason for this different response could be differences in chemical composition and/or structure at the surface of wear debris between the materials. For instance, polystyrene particles with a grafted 2-methacryloyloxyethyl phosphorylcholine layer on the surface were biologically inert with respect to phagocytosis by macrophages.^26^ This different reaction may be due to different proteins adsorbing to the wear particles of the different UHMWPEs, and consequently this may affect uptake of particles by cells. It is well known that the interactions between cells and material are modulated predominantly by the layer of adsorbed proteins on the surface of the material.^27^ At the initial stage of contact between implanted biomaterials and cells, proteins adsorb first and cells adhere to the surface adsorbed proteins within the first few seconds.^28^

It has been reported that oxidised UHMWPE induced granulocyte activation because of serum protein adsorption by its hydrophilic surface.^29^ It is well known that UHMWPE with VE has a lower degree of oxidation before and after γ-ray irradiation^30^ and even after accelerated oxidation treatment.^31^ A certain degree of oxidation must occur on the surface of the UHMWPE during the manufacturing and wear processes. The degree of oxidation occurring during both processes may be reduced significantly by VE addition. It has also been reported that UHMWPE with VE adsorbed slightly less immunoglobulin G (IgG), especially fragments and single heavy chain of IgG, than UHMWPE without VE.^32^ The adsorption of different proteins on the different materials might be different due to lower levels of oxidation in UHMWPE-containing VE. However, UHMWPE particles have been observed inside PBMNCs by confocal laser scanning microscopy in the presence of VE (unpublished results), which indicates that phagocytosis of UHMWPE particles is not affected by the presence of VE.

The mechanisms associated with the reduced cellular responses to wear particles containing VE are not known and require further investigation. VE is essential for human metabolism and is a fat-soluble compound with antioxidant properties, which acts as free radical scavenger. VE also has anti-inflammatory properties, which are well documented.^33^ It has been shown that VE or α-tocopherol decreases the release of reactive oxygen species and decreases the release of proinflammatory cytokines such as TNF-α and IL-1β from monocytes.^34^ Indeed our experiments have shown that VE has successfully mitigated the release of TNF-α in a system containing PBMNCs stimulated with LPS. Also, VE either added as a liquid or when present within the UHMWPE was able to significantly reduce cytokine release.

### Limitations of the present study

The present study focused on the anti-inflammatory potential of VE added to cells, either as a liquid or incorporated into non cross-linked GUR1050 UHMWPE. We investigated a number different possibilities (VE added as a liquid, VE incorporated into conventional UHMWPE and VE-incorporated into highly cross-linked UHMWPE) in order to determine if it was the VE itself that was responsible for the reduction in wear rates and providing beneficial biological outcomes. In addition, the clinical material that was obtained from Japan was not highly cross-linked. Current and future work will concentrate on similar studies using highly cross-linked VE-UHMWPE, which will allow us to determine how the materials differ, with an emphasis on leaching of the VE from the different materials.

## CONCLUSIONS

In conclusion, the virgin UHMWPE and UHMWPE-containing VEs generated comparable wear rates when evaluated using a multidirectional pin on plate wear simulator. Primary human mononuclear cells cultured with wear debris generated from UHMWPE-containing 3000 ppm and 30,000 ppm VE secreted very low levels of osteolytic cytokines, including TNF-α, comparable to the cell only negative control group. However, particles from the virgin material caused the release of significantly higher levels of osteolytic cytokines at comparable volume doses. These results were confirmed when particles from the clinically dosed material, GUR1050 containing 1000 ppm VE were cultured with PBMNCs. Together these results indicate that VE-containing polyethylene has a lower osteolytic potential compared to conventional UHMWPE, which may lead to longer lasting total joint replacement components that may be suitable for younger and more active patients.
